# The role of toxic stimuli combinations in determining safe exposure limits

**DOI:** 10.1016/j.toxrep.2018.10.010

**Published:** 2018-11-07

**Authors:** Ronald N. Kostoff, Marina Goumenou, Aristidis Tsatsakis

**Affiliations:** Research Affiliate, School of Public Policy, Georgia Institute of Technology, Gainesville, VA, 20155, United States; Center of Toxicology Science & Research, Medical School, University of Crete, Heraklion, Greece

**Keywords:** Synergetic effects, Combined effects, Additive effects, Safe exposure limits, Combination toxicity, Joint toxicity, Cumulative risk assessment

## Abstract

This editorial addresses the effects of toxic stimuli combinations on determination of safe Exposure Limits. Examination of thousands of Medline abstracts showed typically that combinations of toxic stimuli can produce damage even when the exposure level of each member of the combination is less than the lowest exposure level of the member that produced damage when tested in isolation. The synergy of the toxic stimuli in combination means less of each component stimulus is required to cause damage compared to exposure levels when tested in isolation. This Editorial concludes there is no reason to believe today that the Exposure Limits on potentially toxic stimuli that have been set by the regulatory agencies are fully protective against serious adverse health effects in all real life exposure scenarios. The conclusion is applicable to essentially all potential contributing factors to disease amenable to Exposure Limits, including not only chemicals but other types of exposures such as radiofrequency radiation (RFR).

## 1 Introduction

Since the dawn of the Industrial Age, and especially over the past century, many thousands of technologies and their products have been introduced to our society. There has been continual concern about the safety of these products, as reflected in their potential adverse impacts on human health. As a result, a number of regulatory agencies have been established for the purpose of ensuring these technology products are safe.

There are three main obstacles these agencies face in determining the degree to which Exposure Limits are protective:

• Sufficiency of existing data for setting safe exposure limits (Has adequate research been done and reported on the toxic stimulus in question and does the research that has been conducted and reported reflect real-world exposures?)
• Sufficiency of incorporating relevant existing data from the biomedical literature
• Trustworthiness of existing data in the biomedical literature [1].

This editorial focuses on the issue of how well real-world exposure effects are reflected by the published literature. The other issues are addressed in part in a recent monograph on occupational exposure permissible limits [2].

The mechanisms used by these agencies to determine safety have been of two main types: laboratory experiments (mainly on animals) and epidemiology studies (mainly on humans). By far, the dominant approach for safety determination of potentially toxic substances has been single stressor studies, mainly on animals [3], [4], [5]. However, many biomedical studies have shown that *combinations* of stressors can enhance the adverse effects of any one of their constituents (relative to its effects when acting in isolation) [6], [7], [8], [9], [10]. Only a relatively few combinations of potentially toxic stimuli decrease the adverse effects of any constituent.

For contributing factors, stimuli combinations typically allow less of each component to cause damage compared to the levels obtained when examining the toxicity of each component in isolation (single stressor experiment, for assessing damage of the stimulus). Thus, when setting safe Exposure Limits for a given combination based on results from experiments in isolation (single stressor), the values used could be substantially higher than those at which that component could cause damage when used in the combination. This has already been proven in a recent study that showed the administration of a mixture of thirteen different chemicals at doses below the individual NOAELs induced biochemical, hematological and redox status changes in rats [11].

## 2 Examples

The following examples show some of these multi-stressor combinations, and the resultant enhancement of adverse effects. For examples 1–3, each of the items tested in isolation was essentially benign (in the parameter range selected), yet in combination contributed to harmful effects. Depending on the exposure levels where each substance in isolation starts to show damaging effects, the difference in setting Exposure Limits based on experiments in isolation (single stressor experiments) and based on the actual experiments in combination could be substantial.

1. “Synergistic toxicity produced by mixtures of biocompatible gold nanoparticles and widely used surfactants” [12]. These mixtures produced synergistic toxicity at concentrations where the individual components were benign.
2. “Synergistic action of the nephrotoxic mycotoxins ochratoxin A and citrinin at nanomolar concentrations in human proximal tubule-derived cells” [13] Only concurrent but not individual exposure to OTA and CIT at nanomolar concentrations led to (i) an increase of TNF protein and mRNA, (ii) a decrease of COX-2 protein and mRNA, (iii) a decrease of E-cadherin protein and (iv) an increase of vimentin and alpha-SMA protein.
3. “DNA damage in rat lymphocytes treated in vitro with iron cations and exposed to 7 Mt magnetic fields (static or 50 Hz)” [14]. Lymphocyte exposure to MF at 7mT did not increase the number of cells with DNA damage in the comet assay. Incubation of lymphocytes with 10 μg/ml FeCl2 did not produce a detectable damage of DNA either. However, when the FeCl_2_-incubated lymphocytes were simultaneously exposed to 7 mT MF the number of damaged cells was significantly increased and reached about 20% for static MF and 15% for power frequency MF.

Examples 4–8 show modest damage from each component of the combination in isolation (in the parameter range selected), but the enhancement afforded by the combination increases the damage substantially.

4. “Concurrent administration of diethylhexyl phthalate reduces the threshold dose at which bisphenol A disrupts blastocyst implantation and cadherins in mice” [15]. Mice were exposed to combinations of BPA and DEHP in doses below the threshold necessary to disrupt implantation on their own. There were fewer normally-developed implantation sites and more underdeveloped implantation sites in females given the combined subthreshold doses, and uterine epithelial cadherin was significantly reduced by these combined doses, but not by the individual doses.
5. “Stress lowers the threshold dose at which bisphenol A disrupts blastocyst implantation, in conjunction with decreased uterine closure and e-cadherin” [16]. Female mice were exposed to rat proximity-induced stress and subthreshold doses of BPA. The combination of rat-exposure stress and BPA significantly disrupted implantation and increased uterine luminal area, whereas either manipulation on its own did not.
6. “Synergistic toxicity of ZnO nanoparticles and dimethoate in mice: Enhancing their biodistribution by synergistic binding of serum albumin and dimethoate to ZnO nanoparticles” [17]. Although nanoZnO had low toxicity to mice, co-exposure to nanoZnO and DM significantly enhanced DM-induced oxidative damage in the liver.
7. “Adverse effect of combination of chronic psychosocial stress and high fat diet on hippocampus-dependent memory in rats” [18]. DTC value for above groups indicated that chronic stress or HFD, alone, resulted in a mild impairment of spatial memory, but the combination of chronic stress and HFD resulted in a more severe and long-lasting memory impairment.
8. “Neurotoxicity induced by methamphetamine-heroin combination in PC12 cells” [19]. These results suggest that the combination of METH and heroin is more neurotoxic than either drug alone.

Example 9 addresses multi-component mixtures. The two takeaways are (1) the enhanced effects predominated at lower effect levels, and (2) the relevance of enhanced effects increased with the complexity of the mixture. So, the greater the number of components, the more important the enhancements, and the lower the levels of some or all of the components required to cause damage.

9. “The synergistic toxicity of the multiple chemical mixtures: Implications for risk assessment in the terrestrial environment” [20]. In four-component and five-component mixtures, the synergistic effects predominated at lower effect levels, while the patterns of interactions found in six, seven, and eight-component mixtures displayed synergism. The relevance of synergistic effects increases with the complexity of the mixture.

Therefore, even the effects of combinations of two contributing factors typically found in laboratory combination experiments may provide insufficiently protective Exposure Limits, compared to the effects of larger combinations characteristic of the real world.

A recent monograph on effects of toxic stimuli combinations [21] presents a much larger number of stressor combination examples and their resultant impacts in its bibliography section. But, even this bibliography is a small fraction of multiple stressor combinations in the biomedical literature.

One disease contributing factor showing a wide gap in Exposure Limits between single stressor studies and combination studies is radiofrequency radiation (RFR). Some RFR combined effects results are shown in [22], [23], and expanded upon in select cases in [21]. Adverse RFR health effects in isolation and in combination are shown in [24], [25].

The effects of RFR combined with one other stressor in lab tests can result in damage at lower RFR exposures than RFR exposures shown to cause serious damage when measured in isolation. The effects of RFR combined with myriad other stressors, as reflected in epidemiology studies, (e.g., [26], [27], [28]) can result in serious damage at RFR exposures orders of magnitude less than RFR exposures shown to cause serious damage when measured in isolation.

## 3 Discussion

The reason few combinations (relative to single stressors) are selected for laboratory studies derives from combinatorics. Given the large number of potential contributing factors to disease [29], the numbers of combinations possible (even with relatively few members per combination) that include these potential contributing factors to disease become unrealistically large very rapidly. A recent monograph on toxic stimuli combinations [21] provides the numbers of combinations possible for different parameter selections.

For example, consider the numbers of combinations required to examine the effect of determining Exposure Limits for RFR (1) measured in isolation vs (2) measured in combination. Assume there are 100 candidate potential contributing factors (e.g. chemicals) whose combination with RFR could result in increased adverse health effects. For potential damage enhancements of RFR combined with only *one* other contributing factor (combination of two factors), 100 experiments would be required to cover all 100 contributing factors. For potential damage enhancements of RFR and *two* other contributing factors (a three-factor combination), 4950 experiments would be required. For potential damage enhancements of RFR and *three* other contributing factors (a four-factor combination), 161,700 experiments would be required. Even RFR and one other contributing factor require a large number of experiments, and the two and three other contributing factor scenarios are completely unrealistic in terms of numbers of experiments and available resources required.

If threshold values for toxic stimuli associated with the onset of disease are reduced (when these stimuli act in combinations) compared to their threshold values when acting in isolation, how should these threshold exposure limits be set for constituents of a combination? Again, RFR is used as the example, for illustrative purposes only. The arguments apply to any potentially toxic stimulus in combination for which Exposure Limits need to be set.

It is clear from the above analysis that RFR in combination with other potential disease contributing factors needs to be studied and used as the basis for setting of credible RFR Exposure Limits. Additionally, Exposure Limits for the non-RFR members of the combination should be re-examined for the impact of RFR on their potential for damage. In fact, the safety objective function should be to minimize damage from the *combination* of potential contributing factors, since what will cause most damage to real people in the real world are (usually) combinations of myriad contributing factors. This requires a quasi-global optimization (on a given combination) rather than a local optimization (on any single constituent). A true global optimization over ALL potential combinations of contributing factors would ensure maximal protection for the public.

In the ideal situation for optimization over each combination, a target would be set for the combination based on ‘acceptable’ damage limits (e.g., less than *X* cancers per 10,000, and/or changes in selected biomarkers less than *Y*%, etc.). The Exposure Limits on each constituent would be set, using an iterative process until the target has been met. In the general case for a specified limit on the damage from the combination, the threshold limit value of each constituent in the combination would be a function of the dose/exposure of every other constituent in the combination. Thus, for a pre-specified damage target for the combination, the threshold limit value for each member of the combination would be defined by a surface in multi-dimensional parameter space, where each dimension corresponds to one constituent of the combination. So, if a toxic stimulus occurs in 1000 combinations being used as the database for determining Exposure Limits, and if the threshold limit value of the toxic stimulus is different for each combination, then caution would dictate that the minimum (or near-minimum) threshold limit value for that toxic stimulus over all the 1000 combinations be used for setting protective Exposure Limits.

Unresolved issues at present (for determining safe Exposure Limits) revolve around numbers of combinations to be evaluated, numbers of constituents in each combination, and the operational procedure for defining the threshold limit value surface. The discipline called Cumulative Risk Assessment (CRA), or Cumulative Effects Assessment (CEA), accounts for multiple stressors acting through multiple pathways. It is not clear from some of the CRA/CEA papers exactly how this discipline would address a situation of the scale enumerated in the section on combinatorics in reference [21]. Many/most of these studies address relatively few combinations, with some of the studies examining different stressors from the same general class. CRA/CEA contain the implicit assumption that the (combinatorics constrained) existing data in the biomedical literature relevant to setting of Exposure Limits is fully taken into account when setting Exposure Limits. They also contain the assumption that the existing data in the biomedical literature can be trusted for accuracy and credibility. Both these assumptions may not be valid, for some cases.

There are two main approaches for obtaining required combination data: lab animal (typically rodent) experiments with ‘tight’ controls on the contributing factor exposures, and epidemiological studies on health effects associated with exposures to contributing factors. The former approach has limitations based on species differences between test animals and humans, and sheer numbers of experiments required to approach the combinations reflective of the real-world. The latter approach has limitations based on not knowing the full ‘signature’ of each individual's exposure to potential disease contributing factors.

Despite their limitations, both approaches are useful. The former approach can provide relative impacts of adding contributing factors and observing the decrease in a given contributing factor threshold dose required to initiate serious diseases. It can also provide insight to biological mechanisms. The latter approach can show macro-level results of the adverse impacts of many contributing factors, even though the details of some of these contributing factors are unknown.

As discussed previously, most of these experiments used to determine Exposure Limits involve one ‘stressor’ in isolation (single stressor experiments). Additionally, combination enhancement effects are ubiquitous across contributing factors and their impacts on disease. Therefore, single stressor experiments as the main determinants for Exposure Limits may be insufficient for human health protection from these potentially toxic contributing factors. For this reason, a consensus should be reached for novel methodological approaches simulating real-life exposures [30], [31].

## 4 Conclusions

• Combinations of stressors usually lower the levels of each constituent associated with damage compared to levels of that constituent tested in isolation.
• Exposure to combinations of stressors reflects the real-world.
• Comprehensive testing of these combinations is severely limited by combinatorial considerations.
• A consensus should be reached for novel methodological approaches simulating real-life exposure.
• We will never be able to obtain a true global optimization over all potential combinations of potentially toxic stimuli to minimize adverse combination enhancement effects. However, it is imperative to go beyond the first-order approximation of single stressor experiments for setting Exposure Limits. Higher-order approximations afforded by combined stressor experiments will provide more realistic Exposure Limits for damage control.
